# Prefrontal Cortex Recruitment to Food Stimuli Differs in Overweight/Obesity and Binge Eating Disorder

**DOI:** 10.1002/erv.70020

**Published:** 2025-08-06

**Authors:** Jennifer Svaldi, Betti Schopp, Philipp A. Schroeder, Dustin Werle, Ann‐Christine Ehlis

**Affiliations:** ^1^ Department of Clinical Psychology and Psychotherapy University of Tuebingen Tübingen Center of Mental Health (TüCMH) Tübingen Germany; ^2^ German Center of Mental Health (DZPG) Tübingen Germany; ^3^ Department of Psychiatry and Psychotherapy University of Tuebingen Tübingen Center of Mental Health (TüCMH) Tübingen Germany

**Keywords:** binge eating disorder, cognitive control, dorsal pathway, fNIRS

## Abstract

**Background:**

At a phenotypical level, the repeated occurrence of binge eating episodes clearly differentiates individuals with binge eating disorder (BED) from individuals with overweight but without BED. Their neural profiles during food‐related inhibition, however, indicate prefrontal hypoactivation in both groups. The present study investigated differential neural activations in upper lateral (right dorsolateral prefrontal cortex [DLPFC]) and inferior (right inferior frontal gyrus [IFG]) control regions during food‐related inhibition by functional near‐infrared spectroscopy (fNIRS). In addition, activity in reward‐related brain regions (orbitofrontal cortex [OFC]) during stimulus processing was measured.

**Method:**

Individuals with BED (*n* = 32), a control group of individuals with overweight and without BED (OWC, *n* = 21), and a control group of individuals with normal weight and without BED (NWC; *n* = 31) underwent a Go/No‐Go (GNG) and a stimulus degradation task during fNIRS, both with food and non‐food stimuli in counterbalanced order.

**Results:**

Most predicted outcomes were not significant. Neural recordings during GNG underscore prefrontal hypoactivation in both BED and OWC relative to NWC, however, with differential alterations: In the Food‐NoGo condition, the BED group displayed hypoactivation in the IFG, while the OWC showed hypoactivation in the DLPFC.

**Conclusions:**

The results suggest differential requirements for DLPFC and IFG recruitment during food‐related inhibition in individuals with BED and overweight.

## Highlights

- Tested neural inhibitory profiles to food stimuli in patients with binge eating disorder (BED) against overweight (OWC) and normal‐weight controls (NWC)
- Optical imaging shows reduced lateral prefrontal activation in OWC relative to BED and reduced inferior frontal activation in BED relative to OWC
- A more specific role of inferior frontal gyrus in response inhibition could indicate the increased inhibitory demand in BED associated with food

## 1 Introduction

Binge eating disorder (BED) is associated with an increased risk of obesity (Villarejo et al. 2012), a substantially reduced quality of life and a significant impairment in regard to physical and mental health (Agh et al. 2015; Mitchell 2016; Wilfley et al. 2016). Although psychological treatments have shown to be efficacious (e.g., Berking et al. 2022; de Zwaan et al. 2017), approximately half of those who undergo such treatments are unable to achieve abstinence from binge eating. Therefore, additional research is needed to identify maintaining factors of BED to facilitate the development of more efficacious and sustainable treatments.

From a neural perspective, both the dorsal and the ventral pathway, which are integral components of the core eating network, interact to influence eating behaviour (Chen et al. 2016). Of course, reward processing and inhibitory responding are to a certain degree interdependent. For instance, for a person with an abnormally strong reward response to food, inhibition of food intake may be more difficult than for a person with a lower reward response. Likewise, a highly impulsive person may be more susceptible to primary rewards such as food than a more controlled individual. Thus, even though reward sensitivity and inhibition are in reciprocal relation (and thus, may be co‐activated at a neural level), they are nonetheless associated with distinct regions and processes from a neurophysiological perspective.

At a behavioural level, this increased food‐related incentive salience is evident in a food‐related attentional bias in individuals with BED relative to overweight and normal weight controls without BED (Deluchi et al. 2017; Popien et al. 2015; Schag et al. 2013; Schmitz et al. 2015, 2014; Werle, Sablottny, Ansorge, et al. 2024). At the experiential level, this is evident in increased levels of hunger, craving, and arousal (Biehl et al. 2019; Castellanos et al. 2009; Nijs et al. 2008, 2009; Nijs et al. 2010; Schmitz et al. 2014; Schmitz and Svaldi 2017; Werthmann et al. 2011).

In contrast, tasks assessing response inhibition in individuals with BED have typically been associated with alterations of the dorsal pathway. Specifically, decreased activity in prefrontal control regions (DLPFC, inferior frontal gyrus [IFG]) has been observed in individuals with BED compared to overweight and normal‐weight controls in both a monetary loss task (Balodis, Kober, et al. 2013) and a Stroop task (Balodis, Molina, et al. 2013), as well as a food‐related Go/No‐Go (GNG) task (Hege et al. 2015). At the behavioural level, several studies have demonstrated an increased impulsive behaviour in individuals with BED relative to controls without BED. These studies included tasks that assessed late‐control inhibitory processes, such as the Stop‐Signal Task or the GNG task (Grant and Chamberlain 2020; Manasse et al. 2016; Mobbs et al. 2011; Svaldi, Naumann, et al. 2014). They also included tasks that measured response interference at an earlier processing stage. Examples of these are tasks that employ pictorial priming, the n‐back task, or the recent‐probes task (Svaldi et al. 2015, 2014).

Interestingly, a recent conceptualisation of the inhibitory control network posits that higher inhibitory demand, as indirectly reflected by the reaction times in go‐trials, necessitates the involvement of the upper portions of the prefrontal cortex for successful inhibition, that is right DLPFC, incremental to the right IFG (Gavazzi et al. 2023). From a clinical perspective, heightened inhibitory demand and subsequent recruitment of upper prefrontal cortex may result from stronger food‐responses in BED, possibly following additional emotional or strong reward response. This perspective expands previous conceptualizations of the inhibitory function of the right prefrontal cortex (Aron et al. 2014, 2016) according to which the right IFG executes inhibition in all conditions. Additionally, the middle and superior frontal gyri are thought to be involved in more complex conditions.

In the present study, functional near‐infrared spectroscopy (fNIRS) was applied to obtain ecologically valid measures of BOLD‐based activation patterns in cortical areas of cognitive control (DLPFC activity), response inhibition (DLPFC and IFG activity) and reward processing (OFC activity). Due to its many practical advantages [for example, a higher temporal resolution, feasibility and cost efficiency as compared to fMRI (Val‐Laillet et al. 2015)], fNIRS has been increasingly employed in research on mental disorders (for a review see Ehlis et al. 2014) and is well suited to assess activation in different areas of the frontal lobe—including the OFC, DLPFC and IFG—as documented by numerous previous studies (Ehlis et al. 2016; Ernst et al. 2014, 2013; Kroczek et al. 2015, 2016; Maier et al. 2019).

To the best of our knowledge, only few studies tested the neural correlates of food stimulus perception and inhibition with fNIRS in BED. In one early pilot study (Rösch et al. 2021), prefrontal activation patterns were assessed in participants with obesity (either with or without BED) during passive viewing and response inhibition tasks. Compared to normal‐weight control participants, obese individuals of both groups showed reduced PFC activation across task conditions. Specifically for the BED group, prefrontal hypoactivation was observed within the left PFC (especially IFG), particularly during the GNG task. Furthermore, increased levels of (self‐reported) emotional dysregulation were specifically associated with prefrontal hypoactivity. The second study (Veit et al. 2021), on the other hand, found trait impulsivity to be significantly (negatively) correlated with PFC activation during inhibitory control within a group of individuals with BED. Moreover, eight weekly sessions of an impulsivity‐focused cognitive behavioural group treatment were associated with a significant increase in right prefrontal activation during food‐related response inhibition. A more recent study, however, did not observe PFC activation during a food‐related reappraisal task in a group of BED patients (Parker et al. 2023). In patients with bulimia nervosa with severe loss of control over eating, prefrontal hypoactivation was again observed in a GNG task (Berner et al. 2023).

The aim of the present study was to extend the previous findings by testing activity in reward related (OFC) and prefrontal control regions (DLPFC, IFG) during a food viewing task and a task that assesses late‐control processes with food and non‐food stimuli in a larger sample. We implemented separate food and non‐food blocks in a GNG task to specifically test for differential activations in lateral and inferior prefrontal control regions. To this end, individuals with BED, overweight (OWC) and normal‐weight (NWC) controls without BED performed a stimulus degradation paradigm, which—in contrast to a pure passive viewing task—required participants to indicate by keypress once the presented stimulus started to degrade to ensure that participants engaged with the presented stimuli. Thus, the stimulus degradation task creates a control condition to the GNG and should yield reward‐related activity. We hypothesised that its additional response component could yield a stronger differentiation between groups than in the previous studies with fNIRS (Rösch et al. 2021; Parker et al. 2023). In addition, participants underwent a GNG task, which assesses participants' ability to withhold a response. It was hypothesised that, when confronted with high‐caloric food stimuli, individuals with BED (relative to OWC and NWC) are characterised by an increased activity in reward‐related brain areas, including the OFC, as well as a decreased activity in prefrontal control regions (DLPFC, IFG). Following the account of increased inhibitory demand, the neural implementation of inhibitory control should switch for BED patients towards more DLPFC activity with increasing demand, that is, during food trials. At the behavioural level, we expected these group differences to be evident in a significantly faster identification of stimulus degradation for food relative to neutral stimuli in the BED group relative to controls in the degradation paradigm, and a higher rate of commission errors in the GNG task in the BED group relative to controls.

## 2 Method

### 2.1 Participants

The present study was conducted as part of the baseline assessment of individuals with BED (*n* = 32) prior to randomisation in a comparative randomized controlled trial (ethical vote: 232/2015B01; Werle, Sablottny, Tuschen‐Caffier, et al. 2024). Participants were recruited from a community sample via ads in local newspapers, flyers in doctors' offices, and university e‐mail distribution lists.

Inclusion criterion was the presence of BED according to the Diagnostic and Statistical Manual of Mental Disorders (DSM‐5; American Psychiatric Association 2013). For all groups, inclusion criteria included age > 18 and < 69. Exclusion criteria were a BMI above 45 kg/m^2^, the presence of substance related and addictive disorders, bipolar disorder, current psychosis or schizophrenia, current suicidal ideation, pregnancy, lactation, severe physical illness and eye diseases (due to the eye‐tracking methodology in the RCT), as well as an ongoing psychotherapy or weight reduction programme. The OWC group (*n* = 31) was BMI‐matched to the BED sample, the NWC (*n* = 31) was recruited in a BMI range from 17.5 kg/m^2^ to 24.9 kg/m^2^; see Table 1.

**TABLE 1:** Sample characteristics.

Group	BED (*n* = 32)	OWC (*n* = 21)	NWC (*n* = 31)
Age	42.53 (14.50)	34.43 (14.18)	31.65 (10.47)*
Gender (f/m)	24/8	14/7	23/8
BMI^a^	31.93 (4.74)	29.62 (3.22)	22.02 (1.55)**
BDI‐II^b^	12.11 (9.65)	3.95 (5.74)**	1.88 (2.70)**
EDE‐Q^b^	2.93 (1.20)	0.70 (0.60)**	0.33 (0.42)**
EDE‐Q restraint	2.19 (1.26)	0.76 (1.23)**	0.49 (0.87)**
EDE‐Q eating concern	2.51 (1.44)	0.14 (0.16)**	0.01 (0.04)**
EDE‐Q weight concern	3.30 (1.54)	0.83 (0.70)**	0.32 (0.49)**
EDE‐Q shape concern	3.73 (1.46)	1.07 (0.75)**	0.52 (0.66)**
Number of OBE’s past 3 months	32.00 (18.15)	0**	0**
Percentage comorbid axis I diagnosis	0.47	0.19	0.13
Percentage comorbid axis II diagnosis	0.13	0.10	0.00

Abbreviations: BDI‐II, Beck Depression Inventory‐II; BED, binge eating disorder; BMI, body‐mass‐index; EDE‐Q, Eating Disorder Examination—Questionnaire; f, female; m, male; NWC, normal‐weight control group; OBE, objective binge episodes; OWC, overweight control group.

^a^ Note that both BED and OWC groups theoretically included normal weight (6.3%/0%), overweight (34.4%/52.4%) and obese BMI (59.4%/47.6%, respectively).

^b^ Based on *n* = 72 (BED: *n* = 27, OWC: *n* = 19, NWC: *n* = 26), due to missing questionnaire data.

*p < 0.05; **p < 0.001.

Presence and absence of a (lifetime) eating disorder was established by means of the Eating Disorder Examination (Hilbert et al. 2004). All other diagnoses were determined by the Structured Clinical Interview for DSM‐IV I (Wittchen et al. 1997) and Axis II (Fydrich et al. 1997).

### 2.2 Materials

#### 2.2.1 Questionnaires

The following questionnaires were employed to characterise the tested samples: (1) The *Eating Disorder Examination Questionnaire* (EDE‐Q; Hilbert and Tuschen‐Caffier 2016) assesses eating pathology over the past four weeks by 28 items (internal consistency *α* = 0.97) (2) The *Beck Depression Inventory‐II* (BDI‐II; Hautzinger et al. 2006) measures severity of depressive symptoms over the past two weeks by means of 21 items (*α* = 0.94) (3) The *Food Cravings Questionnaire‐State* (*FCQ‐S*) *questionnaire* (Meule et al. 2012) measures momentary food craving intensity by three subscales: *hunger* (*α* = 0.88), *reinforcement* (*α* = 0.92) and *desire* (*α* = 0.94; see supplementary results).

#### 2.2.2 Experimental Task 1—Inhibitory Control (Food‐Related Go/NoGo)

**FIGURE 1:**
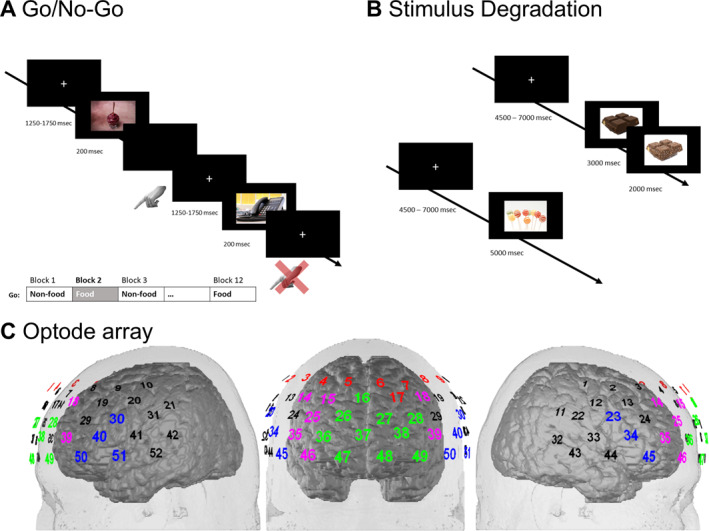
(A) Go‐/No‐Go task in block design with alter task blocks. Food and non‐food stimuli were presented as Go stimulus in half of the blocks. The figure shows two example trials from block 2 (Food‐Go). (B) Food‐related degradation task. Participants had to respond to picture degradation (top trial) as quickly as possible, whereas no response was to be given without degradation (bottom trial). (C) Optode array with 52 measurement channels placed with the middle optode (between channel 47 and 48) over Fpz, the green channels represent the OFC, blue channels the IFG, purple channels the DLPFC‐BA46 and red channels the DLPFC‐BA9.

A visual Go/NoGo task (see Figure 1A) with food and non‐food stimuli was conducted in a block design. Picture size was 300 × 500 pixels and all pictures were matched in luminance. Food stimuli were acquired from Shutterstock (Shutterstock Inc., New York, NY, USA) and consisted mostly of sweets (e.g., chocolate bars, ice cream, donates) and savoury food (e.g., French fries, pizza). Neutral stimuli consisted of office supplies.

Depending on the task block, participants were instructed to react as quickly as possible to either food‐ or non‐food stimuli by keypress, and correspondingly suppress responses to the respective other stimulus category. Task blocks were presented in an alternating fashion (six blocks with food cues as Go stimulus and six blocks with non‐food cues as Go stimulus), with the first condition of a session (Food‐Go or Non‐Food‐Go block) counterbalanced across participants. Block length was approximately 60 s, with 29 Go and 12 NoGo stimuli included in each block (stimulus presentation time: 200 ms; total task duration: 19–20 min). Blocks were separated by 30‐s rest segments.

#### 2.2.3 Experimental Task 2—Attentional Vigilance (Food‐Related Degradation Task)

For the degradation paradigm (see Figure 1B), a total of 120 pictures (60 high‐calorie food pictures, 60 non‐food‐related pictures of office supplies) were presented across two experimental blocks with a self‐paced break (in addition to two practice stimuli; total task duration: approximately 20 min). Participants were instructed to respond as quickly as possible (via button press on a computer keyboard) whenever a picture degraded before the end of the presentation time (50% of all stimuli). Thereby, non‐degrading pictures were presented for 5000 ms, whereas degrading stimuli were fully depicted for 3000 ms before they ‘dissolved into pixels’ for another 2000 ms. The intertrial interval was jittered between 4500 and 7000 ms.

### 2.3 Data Recording (fNIRS)

FNIRS is an optical imaging technique that allows for assessments of the BOLD response in cortical tissue with relatively high ecological validity (relatively low propensity for movement‐related artifacts, no acoustical noise, no restrictive measurement environment). For the measurement, light from the near‐infrared range (690–950 nm) is emitted onto the scalp and penetrates biological tissue (‘optical window’) to reach the upper layers of the cortex. In brain tissue, near‐infrared light is mainly absorbed by the two chromophores oxygenated (O_2_Hb) and deoxygenated haemoglobin (HHb), which differ in their absorption spectrum in the NIR range. Based on the amount of absorbed versus reflected NIR light, both chromophores can therefore be quantified using a modified Beer‐Lambert‐Law (Obrig and Villringer 2003). For the present study, we used a stationary, multi‐channel NIRS system (ETG‐4000, Hitachi Medical Co.) employing light of two wavelengths (695 and 830 nm) and a fixed inter‐optode distance of 3 cm. Measurement channels were defined as the midway point between light emitters and all adjacent detectors. The optode array was placed over bilateral fronto‐temporal areas of the head, with a total of 52 measurement channels (see Figure 1C; the middle optode of the bottom row was placed on position Fpz and that row was oriented towards T3/T4, based on the international 10‐20‐system of electrode placement). Relative concentration changes of O_2_Hb and HHb (mmol*mm/L) were continuously recorded with a sampling rate of 10 Hz.

### 2.4 Data Preprocessing (fNIRS)

NIRS data were analysed using Matlab R2020a (The MathWorks Inc., Natick, USA). For preprocessing, high‐amplitude movement artifacts were corrected using the TDDR algorithm (Fishburn et al. 2019). Afterwards, the correlation‐based signal improvement (CBSI) method was employed to further reduce noise in the data and obtain a ‘true O2Hb’ signal, based on the assumed negative correlation between O_2_Hb and HHb data (Cui et al. 2010). Furthermore, a bandpass filter was applied (0.01–0.1 Hz) and the preprocessed signals were checked again for amplitudes exceeding ± 0.3 mmol*mm/L as well as not‐a‐number (*NAN*) channels containing unusual (infinite) values (in which case they were interpolated). Finally, a Gaussian Kernel filter was applied with a standard deviation of *α* = 0.40 to reduce global signal components due to physiological processes such as breathing. After z‐standardisation, baseline correction (5 s pre‐stimulus/‐block) and linear detrending (−5–80 s), mean values and regression parameters were then calculated individually for each measurement channel, task and experimental condition.

For the analysis of the Go/NoGo block‐design data, mean amplitudes were extracted between 5 and 45 s of all task blocks for statistical analyses. As the degradation paradigm followed an event‐related (instead of a block) design, we used a model‐based analysis (after the same pre‐processing described above) in which event‐related haemodynamic responses were modelled by a canonical BOLD response (peak time: 7 s) and which has been adopted for fNIRS from standard fMRI analyses using the general linear model (GLM) approach (Plichta et al. 2007). Beta values were extracted per subject and condition instead of mean amplitude data. As cortical regions of interest (ROIs), we focused on the dorsolateral prefrontal cortex (DLPFC; BA 9 and 46) and inferior frontal gyrus (IFG; BA 44 and 45) for the well‐established Go/NoGo task. For the degradation paradigm, we included the orbitofrontal cortex (OFC; BA 10 and 11)—as cortical representative of the reward system—as an additional ROI. ROIs were calculated by averaging (per individual and condition) across all channels within the DLPFC–BA 9 (right: channels #2–5; left: channels #6–9 and 17), DLPFC–BA 46 (right: channels #14, 15, 25, 35 and 46; left: channels #18, 39 and 50), IFG (right: channels #23, 34 and 45; left: channels #30, 40, 50 and 51), and OFC/frontopolar area (right: channels #16, 26, 36, 37 and 47; left: channels #27, 28, 38, 48 and 49; see Figure 1C).

### 2.5 Statistical Analyses

For the power analysis see the supplement. We employed analyses of variances (ANOVAs) with repeated measurements with the factors Time (pre‐vs. post‐measurement) and Group (BED, OWC, NWC), which were followed‐up on using *t*‐tests. Degrees of freedom were corrected when necessary due to violations of assumptions. For subjective craving, all subscales were tested (*hunger*, *reinforcement* and *desire*). For behavioural data of the GNG task, Task Condition (Food‐Go vs. Food‐NoGo blocks) was further included for analyses of reaction times and error rates. Further, separate ANOVAs for the fNIRS ROIs with Hemisphere (right vs. left) as an additional within‐subjects factor were conducted. Similarly, for behavioural data of the degradation paradigm, Task Condition (food vs. non‐food stimuli) was included for analyses of reaction times to degrading stimuli, rate of correct hits [to degrading stimuli], and rate of correct rejections [non‐degrading stimuli]. Further, separate ANOVAs for the fNIRS ROIs with Degradation Condition (degrading = targets vs. non‐degrading stimuli) and Hemisphere (right vs. left) as an additional within‐subjects factor were conducted.

We reported standardized effect sizes for (ANOVAs: partial eta squared, *t*‐tests: Cohen's *d*). Two‐sided testing was applied throughout. In order to account for the alpha‐error inflation due to testing effects on multiple outcomes, the significance threshold for the behavioural data was set to 0.025 (GNG: two outcomes) and to 0.0167 (Degradation paradigm: three outcomes). For the three scales of the FCQ as well as fNIRS data stemming from different cortical ROIs, we applied a modified Bonferroni approach, considering the dependency of the outcome measures of each domain in terms of Pearson correlations. For the FCQ‐S data, this modified Bonferroni approach according to Dubey and Armitage‐Parmar (D/AP; as cited in Sankoh et al. 1997; and automatised on the www.quantitativeskills.com/sisa/calculations/bonfer.htm webpage) resulted in a corrected, critical *p*‐value of 0.04 (with an average correlation between the three scales of *r* = 0.802). For the fNIRS GNG, the D/AP‐corrected critical *p*‐values was 0.022 for three ANOVAs and an average correlation between the different ROIs of *r* = 0.256 (calculated separately for Food‐Go and Food‐NoGo blocks, then averaged across both). Finally, for the fNIRS data from the degradation paradigm, a mean correlation between the different ROIs of *r* = 0.43—combined with a total number of four ANOVAs—led to a D/AP‐corrected significance threshold of *p* < 0.0227.

## 3 Results

### 3.1 Go/NoGo Task

#### 3.1.1 Behavioural Data

Due to aberrant behavioural data (responses independent of the task instruction), three participants of the BED group had to be excluded from the analysis of the Go/NoGo data (new BED group size: *n* = 29; age: 43.07 ± 14.58 years; BMI: 31.63 ± 4.75). Similarly, four single blocks were excluded in different participants (two BED, one OWC, one NWC) due to erroneous responses on all trials. For reaction times to food pictures (‘Food‐Go blocks’) and non‐food pictures (‘Food‐NoGo blocks’; see Table 2), a repeated measures ANOVA showed a significant main effect of Task Condition (*F*_1, 78_ = 27.982, *p* < 0.001, ƞ_p_^2^ = 0.264) with faster responses to food as compared to non‐food stimuli across groups. There was no significant interaction of Task × Group (*F*_2, 78_ = 0.215, *p* = 0.807, ƞ_p_^2^ = 0.005). For error rates (i.e., button presses following food pictures in ‘Food‐NoGo blocks’ and following non‐food pictures in ‘Food‐Go blocks’; see Table 2), the ANOVA also indicated a significant main effect of Task Condition (*F*_1, 78_ = 64.430, *p* < 0.001, ƞ_p_^2^ = 0.452), with more false alarms (i.e., NoGo errors) for food‐compared to non‐food stimuli across groups. The main effect group (*F*_2, 78_ = 0.410, *p* = 0.665, ƞ_p_^2^ = 0.010) and the interaction of both factors was again non‐significant (*F*_2, 78_ = 0.623, *p* = 0.539, ƞ_p_^2^ = 0.016).

**TABLE 2:** Reaction times and number of false alarms for the Go/NoGo task.

		RT (ms)		False alarms	
	n	Food‐Go	Non‐food‐Go	Food‐NoGo	Non‐food‐ NoGo
OWC	21	438.1 ± 43.2	452.6 ± 50.4	12.7 ± 7.4	8.1 ± 6.9
NWC	31	439.3 ± 46.2	450.5 ± 39.2	10.9 ± 7.4	5.5 ± 4.5
BED	29	448.1 ± 32.9	459.1 ± 39.3	10.0 ± 5.8	6.0 ± 5.5

*Note:* Mean ± standard deviation of reaction times (RT) to Go stimuli (in ms) and the number of unsuppressed responses (i.e. false alarms) to Food‐NoGo and Non‐food‐NoGo stimuli for overweight controls (OWC), normal weight controls (NWC) and binge‐eating disorder (BED) patients.

#### 3.1.2 fNIRS Data

**FIGURE 2:**
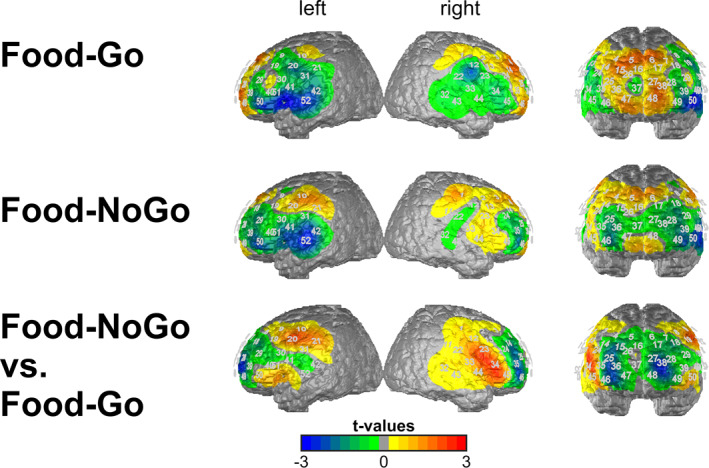
Group contrasts (t‐values) of CBSI‐corrected O_2_Hb data (averaged amplitudes 5–45 s after stimulus onset) of the OWC versus BED group for Food‐Go and Food‐NoGo blocks of the Go/NoGo task as well as the difference between both conditions (Food‐NoGo minus Food‐Go). Negative values (blue) mark areas with reduced activation in the OWC group (NoGo‐specific in the DLPFC), positive values (red) mark areas with reduced activation in the BED group (NoGo‐specific in the IFG).

The 2 × 2 × 3 ANOVA (Condition × Hemisphere × Group) for BA 46 of the DLPFC revealed a significant main effect of Hemisphere (*F*_1, 78_ = 15.352, *p* < 0.001, η_p_^2^ = 0.164) with higher amplitudes for the right than the left hemisphere. Furthermore, a marginally significant interaction of all three factors (*F*_*2*_, 78 = 3.784, *p* = 0.027, η_p_^2^ = 0.088) reflected reduced left‐hemispheric activation during Food‐NoGo blocks in the OWC compared to both the BED (*t*_48_ = 2.300, *p* = 0.026, *d* = 0.511) and NWC group (*t*_50_ = 2.310, *p* = 0.025, *d* = 0.547). Moreover, for the difference measure of Food‐NoGo‐minus‐Go, the OWC group again showed reduced activation compared to the BED group, this time for the right hemisphere only (*t*_48_ = 2.053, *p =* 0.046, *d* = 0.634) (see also Figure 2).

The 2 × 2 × 3 ANOVA (Condition × Hemisphere × Group) for the IFG again revealed a significant interaction of all three factors, but only at an uncorrected significance level (*F*_2, 78_ = 3.378, *p* = 0.039, η_p_^2^ = 0.080), with—this time—significantly reduced Food‐NoGo‐specific activation over the right IFG within the BED compared to the OWC group (difference measure of Food‐NoGo‐minus‐Go; *t*_48_ = 2.594, *p* = 0 013, *d* = 0.642; see Figure 2). This group effect in the difference measure was due to a tendency of OWC controls to show higher amplitudes for Food‐NoGo compared to Food‐Go blocks (putatively reflecting increased inhibitory control when responses to food pictures had to be suppressed; *t*_20_ = 1.879, *p* = 0.075, *d* = 0.626), whereas BED patients showed an opposite tendency, with reduced activation during Food‐NoGo compared to Food‐Go blocks (*t*_28_ = 1.818, *p* = 0.080, *d* = 0.654). The NWC group showed no significant difference between both conditions, indicating comparable levels of inhibitory control during blocks were responses to food versus non‐food pictures had to be suppressed.

For BA9 of the DLPFC no significant main effects or interactions emerged (all *F*s < 2.70, *p*s > 0.100, η_p_^2^ ≤ 0.035). Similarly, there were no significant main effects or interactions for the OFC/frontopolar cortex (all *Fs* < 2.73, *ps* > 0.072, η_p_^2^ ≤ 0.065).

### 3.2 Degradation Paradigm

#### 3.2.1 Behavioural Data

Detailed behavioural results are reported in the Supporting Information S1 and summarised here. In brief, degrading food pictures were faster responded to than degrading non‐food pictures across groups (*F*_1, 76_ = 22.249, *p* < 0.001, ƞ_p_^2^ = 0.226). Non‐degrading stimuli were less often correctly rejected (averaged across both conditions) in the OWC (27.93 ± 2.79) compared to the NWC group (30.34 ± 2.73; *t*_47_ = 3.029, *p* = 0.004, *d* = 0.874) and—with marginal significance—also compared to the BED group (29.47 ± 2.68; *t*_49_ = 1.982, *p* = 0.053, *d* = 0.564), in a main effect of Group for correct rejection (*F*_2, 76_ = 4.713, *p* < 0.0167, ƞ_p_^2^ = 0.110). For correct responses, a marginal significant interaction between Condition and Group (*F*_3, 76_ = 3.308, *p* = 0.042, ƞ_p_^2^ = 0.080) indicated that BED patients showed a reduced number of hits compared to OWC controls, but only to non‐food stimuli (28.40 ± 3.59 vs. 30.57 ± 3.83 hits; *t*_49_ = 2.070, *p* = 0.044, *d* = 0.589). Within‐group comparisons further revealed a significant difference between conditions only within the BED group, with a higher number of correct responses to food (29.27 ± 3.05) compared to non‐food stimuli (28.40 ± 3.59; *t*_29_ = 3.024, *p* = 0.005, *d* = 0.552). There were no further significant main effects or interactions.

#### 3.2.2 fNIRS‐Data

To analyse the fNIRS data derived from the degradation paradigm in a total of 80 participants (*n* = 31 BED, *n* = 21 OW, *n* = 28 NW; see above), 2 × 2 × 2 × 3 ANOVAs were calculated separately for the different ROIs, with the within‐subjects factors Food Condition (food vs. non‐food stimuli), Degradation Condition (degrading = targets vs. non‐degrading stimuli) and Hemisphere (left vs. right) as well as the between‐subjects factor Group (BED vs. OWC vs. NWC).

For BA46 of the DLPFC, we found a significant main effect of Degradation Condition, with larger beta values (i.e., activation) for degrading stimuli, that is, in the target condition (*F*_1, 77_ = 17.007, *p* < 0.001, ƞ_p_^2^ = 0.181). A significant interaction Degradation Condition × Hemisphere (*F*_1, 77_ = 7.427, *p* < 0.01, ƞ_p_^2^ = 0.088) further indicated that this effect was stronger for the right (difference between degrading minus non‐degrading stimuli: 9.893 ± 19.678) than the left (corresponding difference measure: 4.386 ± 14.345) hemisphere (*t*_79_ = 2.809, *p* = 0.006, *d* = 0.314). There were no significant interactions with group (*F*s < 1.40, *p*s > 0.250, ƞ_p_^2^ ≤ 0.035).

Regarding reward‐related cortical activity, similarly, the OFC/frontopolar cortex data also revealed a significant main effect of Degradation Condition, with again higher beta values for degrading (i.e., target) stimuli (*F*_1, 77_ = 10.076, *p* = 0.01, ƞ_p_^2^ = 0.116); these data are, however, difficult to interpret since negative beta values were found for both conditions, indicating cortical ‘deactivation’ in this part of the brain (that was—accordingly—less pronounced for target stimuli). There were no significant interactions with group (*F*s < 2.90, *p*s > 0.06, ƞ_p_^2^ ≤ 0.069).

For the IFG, no significant main effects or interactions occurred (all *F*s < 2.70, *p*s > 0.10, ƞ_p_^2^ ≤ 0.033).

For BA9 of the DLPFC, no significant effects occurred (all *F*s < 3.20, *p*s > 0.05, ƞ_p_^2^ ≤ 0.04).

## 4 Discussion

The present study tested the neural implementation of inhibitory control with two behavioural paradigms and fNIRS in binge eating disorder and two control groups. For the GNG task, we hypothesised that higher demand in trials requiring food‐related inhibition would activate the lateral portions of the right prefrontal cortex to achieve successful inhibition and would differentiate the clinical from the control groups. For the degradation paradigm we assumed an increased activity in reward‐related brain areas (OFC) and decreased in prefrontal control regions, which, at the behavioural level would be evident in a facilitated identification of food pictures relative to neutral pictures in the BED relative to the control groups.

Our results replicated inhibitory control‐related prefrontal hypoactivation in participants with overweight and binge eating disorder, albeit with several interesting alterations. Hypoactivation was evident in the DLPFC of the overweight control group, although the lateralisation and food‐specificity was not clear in the results, likely due to a relatively small effect size. In contrast, hypoactivation in the IFG in the food‐related no‐go condition was evident in patients with BED, with an opposite descriptive pattern in individuals with overweight and obesity. This pattern is conclusive with a comparably previous study in BED (Rösch et al. 2021) and with the decrease following response inhibition training (Veit et al. 2021). Against the recent theoretical suggestion of the auxiliary function of the DLPFC function in inhibitory control (Gavazzi et al. 2023), the differential neural profiles may potentially indicate a higher demand for inhibitory control in BED, which calls for additional recruitment of the upper prefrontal cortical regions. Interestingly, a recent conceptualisation of the inhibitory control network posits that higher inhibitory demand, as indirectly reflected by the reaction times in go‐trials, necessitates the involvement of the upper portions of the prefrontal cortex for successful inhibition, that is right DLPFC, incremental.

In contrast, hypoactivation in the DLPFC with slight activation increases in IFG may already trigger the need for further inhibitory control implementation in a food‐related context. Accordingly, although the present results are very tentative and should not be overstated, especially considering the nonsignificant behavioural findings, they may point to neural alterations in BED relative to OWC relating to the upper‐portion prefrontal cortex regions and overarching implementation of cognitive control. Functionally, this could indicate that patients with BED recruit globally, potentially to attempt restrained eating, to compensate a stronger reward response (note however that no difference was found in the OFC activation to food stimuli in the present study) or to compensate for inhibition‐specific deficits in a non‐binging experimental context, whereas OWC still rely on the right IFG as the specific execution of inhibitory control, although its activity is potentially not sufficient in the eating‐related context. In turn, the additional recruitment of inhibitory areas in BED could be also related to their reduced number of hits to non‐food stimuli, compared to OWC. Noteworthy, this assumption requires further investigation and additional behavioural findings, possibly also including other reward‐related regions.

Consistent with passive viewing paradigms of previous research (Rösch et al. 2021; Parker et al. 2023), we also did not observe group differences in reward‐related regions during the degradation paradigm in the present research. At a neural level, these results confirm that alterations in prefrontal cortex activation are related to task‐specific and food‐related inhibition. The findings also align with inconsistent neural findings on reward sensitivity in differentiating patients with BED from controls with overweight but without BED (Schienle et al. 2009; Weygandt et al. 2012; Rösch et al. 2021; Parker et al. 2023; Kessler et al. 2016). Notably, previous research employing the degradation paradigm has demonstrated an increased vigilance (i.e., faster recognition) for food over neutral stimuli in females with BED (Schmitz et al. 2014). Future studies should therefore test for mediating and moderating factors such as mood (Stein et al. 2007) and the menstrual cycle (Nannt et al. 2024), which both have demonstrated to affect food intake in BED. Beyond this, previous research has shown that repeated behaviour associated with reward (i.e., habit formation) leads to a shift from goal‐directed to habitual behaviour, which is accompanied by a shift in the neural systems supporting behaviour. As such, once a behaviour becomes a habit, it is under control of neural systems associated with the dorsolateral PFC (Graybiel 2008; Balleine and O'doherty 2010; O'Doherty et al. 2004; Daw and Shohamy 2008). Considering the large age‐span of first‐incidence of BED (Preti et al. 2009) on the one hand and its chronicity (Hudson et al. 2007) on the other hand, our sample may have been quite heterogeneous in terms of disorder duration. Disorder chronicity may thus have weakened activation in the OFC and future studies should therefore assess participants' disorder duration to test for this hypothesis. Last but not least, the null findings from this paradigm may also indicate its low sensitivity to food‐related reward activity.

A strength of the present study is the inclusion of two control groups with and without overweight. This design enabled us further investigation of the specificity of prefrontal cortex regions, and the differential requirements for upper DLPFC and lateral IFG recruitment in different manifestations of eating in the absence of loss of control versus binge eating. However, the present study also has several limitations. First, the robustness of our findings hinges on the lack of behavioural results, in particular, no significant group × task interaction in the GNG. In the present implementation of the task, that was optimised for neural recording, behavioural performance in the GNG may not have been sensitive enough to detect such group differences. Moreover, despite testing relatively large groups, several participants dropped out of the analyses due to aberrant behavioural responses, which potentially limits the statistical power of our analyses. As the study was initiated already in 2018, short‐distance correction was not yet implemented in our routines, which potentially limited the signal‐to‐noise ratio and thus the statistical power in our recordings. Moreover, although fNIRS has a better temporal resolution as compared to fMRI, the spatial resolution is limited and pertains to cortical regions only; thus, subcortical hubs of reward sensitivity were not entirely covered. Crucially, fNIRS has been shown to be compatible with behavioural paradigms such as the GNG task and food degradation paradigm. We did not measure full‐coverage activity but focused on inhibitory control regions in the prefrontal cortex, and orbitofrontal cortex, according to our hypotheses. In addition, causal evidence in order to substantiate the proposed theory (Gavazzi et al. 2023) would necessitate the implementation of simultaneous and navigated neuromodulation targeting the (right) DLPFC and IFG.

The lack of behavioural differences precludes any clinical implications at this stage. Further empirical work should first establish the causal role of DLPFC versus IFG regions in food‐related inhibition in OWC and BED, for instance, using spatially optimised neuromodulation. Further longitudinal research is required to investigate the dynamics between possible pre‐existing (neural) differences and learning, for example, incentive sensitisation. Given that inhibitory control is recurrently discussed as a treatment component for both BED and overweight/obesity (De Klerk et al. 2023; Eichen et al. 2023), a more nuanced understanding of the inhibitory demand associated with psychopathology profiles and elicited by food‐related inhibition trainings might enhance their efficacy in future trials. Furthermore, elucidation of the ventral stream with other neural and behavioural paradigms may enable the investigation of reward‐related activities in parallel.

In conclusion, we carefully suggest differential neural profiles underlying food‐related inhibition in individuals with overweight versus binge eating. Contemporary updates on late response inhibition based on meta‐analytic findings from fMRI indicated a distinction between rIFG and rDLPFC regarding the individual complexity of stimuli (Gavazzi et al. 2023). The neural patterns in our results suggested that individuals with BED preferably recruit the upper prefrontal cortex whereas individuals with overweight but without BED rely more on the IFG, which may not suffice for difficult food‐related situations. However, effect sizes are small and more research is required. This finding could relate to the increased emotional complexity food stimuli exert in BED.

## Ethics Statement

The authors assert that all procedures contributing to this work comply with the ethical standards of the relevant national and institutional committees on human experimentation and with the Helsinki Declaration of 1975, as revised in 2008.

## Conflicts of Interest

The authors declare no conflicts of interest.

## Supporting information

Supporting Information S1

## Data Availability

The data that support the findings of this study are available from the corresponding author upon reasonable request.
