# Supplementary material for: Prefrontal Cortex Recruitment to Food Stimuli Differs in Overweight/Obesity and Binge Eating Disorder
Source: Eur Eat Disord Rev. 2025 Aug 6;34(1):202–12. doi: 10.1002/erv.70020 (PMC12694698; doi:10.1002/erv.70020)
Supplement: Supplementary file 1 — Supporting Information S1 [file ERV-34-202-s001.docx]

**Inhibitory demand and prefrontal cortex recruitment to food stimuli differs in overweight/obesity and binge eating disorder**

**SUPPLEMENTARY MATERIALS**

**Supplementary Methods: Procedure**

After signing the informed consent, participants underwent the diagnostic interviews (during which weight and height were registered) and then filled in the questionnaires. They were then invited to three separate laboratory sessions. Session one comprised an eye tracking experiment (Sablottny et al., under review), session two an EEG experiment (Werle, Sablottny, Ansorge, et al., 2024). The two experimental tasks were part of session three and lasted about 90 minutes, including preparation time for the fNIRS assessment and stimulus ratings (see below). Between experimental task 1 and 2, participants had a break of 15 minutes. Following this session, participants were enrolled in the RCT (Werle, Sablottny, Tuschen-Caffier, & Svaldi, 2024).

At the beginning of the fNIRS session, participants filled in the 15-items Food Cravings Questionnaire (Meule et al., 2012). Then, they were prepared for the fNIRS measurement and conducted the experimental paradigms, with task order (degradation or Go/NoGo first) counterbalanced across participants. At the end of the session, participants once again filled in the FCQ.

**Supplementary Methods: Post-hoc power analysis**

After data analysis a sensitivity power analysis was conducted in R / pwr (Champely, 2020), in order to quantify the statistical power for detecting potentially smaller effect sizes than previously reported. For the contrast OWC/BED, with conventional parameters (α = 0.05, 1 – β = 0.8), large effect sizes could be detected (d > .80). A slightly higher sensitivity was available for the contrast HC/BED (d > .72). The power to detect a more moderate effect size of d = 0.626 (i.e., the contrast of IFG Food No-Go Neural activation) within OWC controls was acceptable (1-β = 0.79).

**Supplementary Results: Food Cravings Questionnaire-State (FCQ-S)**

Complete sets of FCQ-S scores before and after the fNIRS measurement were obtained in a total of *n*=75 participants (*n*=30 BED, *n*=17 OW, *n*=28 NW). Repeated-measures ANOVAs (Time × Group) were conducted separately for all three FCQ-S scales (*desire*, *hunger* and *reinforcement*) and revealed significant main effects of Time for all three subscales (14.178 ≤ *F*_1, 72_≤ 34.638, .165 ≤ ƞ_p_^2^≤ .325, all *p* < .001) as well as significant main effects of Group for the *reinforcement* (*F*_2, 72_= 10.149, *p* < .001, ƞ_p_^2^= .220) and *desire* subscale (*F*_2, 72_= 16.515, *p* < .001, ƞ_p_^2^= .314; vs. *hunger*: *F*_2, 72_= 2.422, *p* = .096, ƞ_p_^2^= .063), with no significant interactions (all *F*s < 0.90, *p*s > .400, ƞ_p_^2^< .025). In more detail, scores for all three scales increased significantly across groups from pre- to post-measurement, i.e., after the two food-related paradigms as compared to the pre-measurement baseline. Post-hoc analyses of the significant group effects further revealed higher craving ratings on both the *reinforcement* and the *desire* subscale (averaged across both assessment points) in the BED compared to both the NWC (*reinforcement*: *t*_52.39_= 3.878, *p* < .001, *d* = 0.865; *desire*: *t*_47.76_= 5.222, *p* < .001, *d* = 0.872) and OWC group (*reinforcement*: *t*_44.17_= 3.653, *p* < .001, *d* = 0.882; *desire*: *t*_45_= 4.016, *p* < .001, *d* = 0.914), with no significant difference in FCQ-S scores of either scale between NWC and OWC group (*t*_43_< 1.20, *p*s > .200, *d*s ≤ 0.61).

**Supplementary Table 1 - Food-Craving-Questionnaire pre- and post-measurement**

| Group n= 75 |  | BED (*n* = 32) | OWC (*n* = 20) | NWC (*n* = 30) |
| --- | --- | --- | --- | --- |
| FCQs -hunger | pre-measurement | 2.49 (1.23) | 1.97 (0.79) | 1.74 (0.87) |
| FCQs -reinforcement |  | 2.57 (1.07) | 1.85 (0.58) | 1.73 (0.67) |
| FCQs -desire |  | 2.59 (1.09) | 1.68 (0.62) | 1.43 (0.46) |
| FCQs -hunger | post-measurement | 2.79 (1.24) | 2.43 (0.98) | 2.29 (1.17) |
| FCQs -reinforcement |  | 2.98 (1.03) | 1.94 (0.94) | 2.00 (0.86) |
| FCQs -desire |  | 3.17 (1.17) | 2.07 (0.72) | 1.88 (0.88) |

Table Note. Means and standard-deviations of FCQs subscales hunger, reinforcement and desire for the binge-eating disorder patients = BED, the overweight/obese controls = OWC and the normal-weight controls = NWC

**Supplementary Results: Degradation paradigm, Behavioral data**

Out of the 84 participants included in this study, five had to be excluded from the behavioral analyses of the degradation paradigm: Four participants correctly responded to less than half of the degrading stimuli according to the task instructions; in one participant, the false response button was instructed, so they were excluded from the analysis of the behavioral data but were included in the fNIRS data analysis. This resulted in a final sample size of n=21 OW controls (34.43 ± 14.18 years, 29.62 ± 3.22 BMI), n=30 BED patients (42.48 ± 14.74 years, 31.67 ± 4.57 BMI) and n=28 NW controls (31.86 ± 10.89 years, 22.00 ± 1.60 BMI) for this part of the analysis.

A repeated-measures ANOVA for the reaction times to degrading food- and non-food stimuli (with the between-subjects factor Group) showed a main effect of Condition with faster responses to food- compared to non-food pictures (F1, 76 = 22.249, p < .001, ƞp2 = 0.226). The main effect Group and the corresponding interaction were not significant (F < 1.35, p > .250, ƞp2 < 0.035).

For the number of correct responses to food- and non-food stimuli, we found no main effects of Condition or Group (Fs < 2.40, ps > .100, ƞp2 < 0.06), but – at an uncorrected significance level – a significant interaction between both factors (F3, 76 = 3.308, p = .042, ƞp2 = 0.080). Post-hoc testing revealed that BED patients showed a reduced number of hits compared to OWC controls, but only to non-food stimuli (28.40 ± 3.59 vs. 30.57 ± 3.83 hits; t49 = 2.070, p = .044, d = 0.589). OWC and NWC did not differ significantly in two-sided testing; neither did BED and NWC (all ts < 1.96, ps > .05, d < 0.57). Within-group comparisons further revealed a significant difference between conditions only within the BED group, with a higher number of correct responses to food (29.27 ± 3.05) compared to non-food stimuli (28.40 ± 3.59; t29 = 3.024, p = .005, d = 0.552).

And finally, a repeated-measures ANOVA for the number of correct rejections (i.e., correct “non-button-presses” to non-degrading stimuli) showed no main effect of Condition (F1, 76 = 0.704, p = .404, ƞp2 = 0.009) and no significant interaction (F2, 76 = 0.831, p = .439, ƞp2 = 0.021); however, a significant main effect Group (F2, 76 = 4.713, p < .0167, ƞp2 = 0.110) was found, due to a lower number of correct rejections (averaged across both conditions) in the OWC (27.93 ± 2.79) compared to the NWC group (30.34 ± 2.73; t47 = 3.029, p = .004, d = 0.874) and – with marginal significance – also compared to the BED group (29.47 ± 2.68; t49 = 1.982, p = .053, d = 0.564).
